# Camptothecin and khat (*Catha edulis *Forsk.) induced distinct cell death phenotypes involving modulation of c-FLIP_L_, Mcl-1, procaspase-8 and mitochondrial function in acute myeloid leukemia cell lines

**DOI:** 10.1186/1476-4598-8-101

**Published:** 2009-11-13

**Authors:** Therese Bredholt, Elizabeth AO Dimba, Hanne R Hagland, Line Wergeland, Jørn Skavland, Kjell O Fossan, Karl J Tronstad, Anne C Johannessen, Olav K Vintermyr, Bjørn T Gjertsen

**Affiliations:** 1 The Gade Institute, University of Bergen, Bergen, Norway; 2 Department of Oral and Maxillofacial Surgery, University of Nairobi, Nairobi, Kenya; 3 Department of Biomedicine, University of Bergen, Bergen, Norway; 4 Institute of Medicine, Hematology Section, University of Bergen, Bergen, Norway; 5 Laboratory of Clinical Biochemistry, Haukeland University Hospital, Bergen, Norway; 6 Department of Pathology, The Gade Institute, Haukeland University Hospital, Bergen, Norway; 7 Department of Medicine, Hematology Section, Haukeland University Hospital, Bergen, Norway

## Abstract

**Background:**

An organic extract of the recreational herb khat (*Catha edulis *Forsk.) triggers cell death in various leukemia cell lines *in vitro*. The chemotherapeutics camptothecin, a plant alkaloid topoisomerase I inhibitor, was tested side-by-side with khat in a panel of acute myeloid leukemia cell lines to elucidate mechanisms of toxicity.

**Results:**

Khat had a profound effect on MOLM-13 cells inducing mitochondrial damage, chromatin margination and morphological features of autophagy. The effects of khat on mitochondrial ultrastructure in MOLM-13 correlated with strongly impaired routine respiration, an effect neither found in the khat-resistant MV-4-11 cells nor in camptothecin treated cells. Enforced expression of anti-apoptotic Bcl-2 protein provided protection against camptothecin-induced cell death and partly against khat toxicity. Khat-induced cell death in MOLM-13 cells included reduced levels of anti-apoptotic Mcl-1 protein, while both khat and camptothecin induced c-FLIP_L _cleavage and procaspase-8 activation.

**Conclusion:**

Khat activated a distinct cell death pathway in sensitive leukemic cells as compared to camptothecin, involving mitochondrial damage and morphological features of autophagy. This suggests that khat should be further explored in the search for novel experimental therapeutics.

## Background

In search for novel experimental cancer therapies, we are examining cellular and molecular effects of an organic extract of the recreational herb khat [1,2]. Adverse health effects are associated with habitual khat use, but underlying molecular mechanisms are poorly understood [3]. The botanical alkaloid camptothecin (CPT) induces apoptosis through a defined mechanism in cancer cell lines and its derivatives irinotecan and topotecan are widely used cancer therapeutics [4-6].

Acute myeloid leukemia (AML) is an aggressive hematological malignancy of the myeloid progenitor cells, characterized by a differentiation block and extensive leukemic cell accumulation in the bone marrow [7]. Therapeutic approaches in AML may be opposed by numerous genetic alterations, often affecting pathways regulating apoptosis [8-10]. Identification of novel substances using alternative cell death pathways or capable of restoring sensitivity to apoptosis is therefore of therapeutic importance.

Programmed cell death may occur through the mechanisms of apoptosis, necrosis and excessive autophagy, with the mitochondria playing a central role in its regulation [11,12]. The Bcl-2 family of proteins is involved in regulation of mitochondria-mediated death by affecting the stability of the outer mitochondrial membrane. Anti-apoptotic Bcl-2 is often found over-expressed in AML, mediating therapeutic resistance and poor survival [13,14]. Levels of anti-apoptotic Bcl-2 and pro-apoptotic Bax have been shown to correlate with spontaneous apoptosis in AML cells *in vitro *[10], and the ratio of Bax to Bcl-2 in patient cells is proposed to predict clinical response and outcome [8]. An important role is played by the anti-apoptotic Mcl-1 member of the Bcl-2 protein family, illustrated by its ability to block therapeutic targeting of other Bcl-2-like proteins [15].

Mitochondria participate in cell death induction through release of apoptogenic proteins to the cytosol and generation of excess levels of reactive oxygen species (ROS). The mitochondrial respiratory chain serves as a major source of cellular ROS and also represents a target for its damaging effects [16].

Programmed cell death may be initiated from within the cell (e.g. by DNA damage, ROS, hypoxia), through ligand activation of cell surface death receptors or through a combination of both. The proteolytic inactive procaspase-8 homologue cellular FLICE inhibitory protein (c-FLIP) is an antagonist of receptor-mediated cell death [17,18]. c-FLIP over-expression confers resistance to receptor-mediated apoptosis in various malignancies [19,20] and down-regulation of c-FLIP has been shown to sensitize tumor cells to apoptosis via cell death receptors [21-23].

We have compared khat and CPT side-by-side in selected human AML cell lines in order to evaluate the cell death mechanisms involved. Khat-induced cell death was characterized by adverse effects on mitochondrial structure and function, chromatin margination and morphological features of autophagy, including Mcl-1 down-regulation, c-FLIP_L _cleavage and procaspase-8 activation. In contrast, CPT-induced apoptosis was characterized by nuclear fragmentation and unaffected mitochondrial function.

## Results

### AML cell lines exhibited different sensitivities to khat and CPT

**Figure 1:**
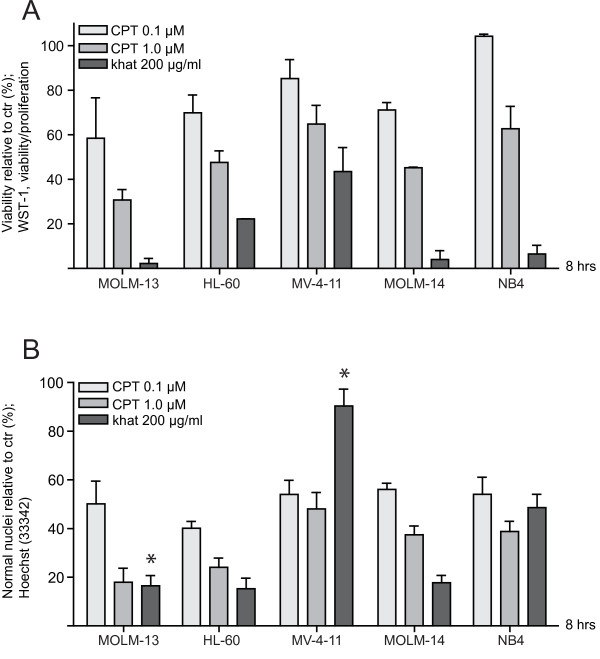
**AML cell lines exhibited different sensitivities to khat and CPT**. Selected human AML cell lines were exposed to 200 μg/ml khat, 0.1 μM CPT and 1.0 μM CPT for 8 hrs and evaluated for toxic effects. **(A)** Effects on cell viability/proliferation were assessed using the WST-1 assay. The results were collected by measuring absorbance (450 nm - 620 nm) and are presented as: [(absorbance treated cells)/(absorbance control cells)] × 100. **(B) **Effects on nuclear morphology were determined following Hoechst staining using epifluorescent microscopy. The results are presented as percentages cells with normal nuclear morphology as compared to controls. The difference in khat sensitivity between MOLM-13 and MV-4-11 was statistical significant as indicated by the asterisk (p < 0.05). The results in A are based on two separate experiments and the results in B based on three separate experiments; all experiments were run in triplicate; the bars represent standard error of mean (SEM).

Selected AML cell lines with molecular features representative of the malignancy (Methods; Table 1) were exposed to 200 μg/ml khat [1,24], and 0.1 and 1.0 μM CPT for 8 hrs before evaluation of toxic effects. When employing a viability/proliferation assay based on mitochondrial activity (WST-1) the monocytic cell lines MOLM-13 and MOLM-14 and the promyelocytic NB4 cell line were observed to be most sensitive to khat. The biphenotypic MV-4-11 cell line was the most resistant, particularly to khat (Fig. 1A).

**Table 1:** Endogenous Bcl-2 and Bax protein levels (MFI ± SD); selected molecular characteristics.

**Cell line**:	**Bcl-2**:	**Bax**:	**Bcl-2/Bax**:	*TP53*:	*FLT3*:
MOLM-13	43.4 ± 15.7	12.8 ± 6.1	3.6 ± 0.7	wt	ITD
HL-60	83.5 ± 13.3	27.1 ± 3.9	3.1 ± 0.6	del	wt
MV-4-11	36.4 ± 10.7	13.0 ± 1.7	2.8 ± 0.5	wt	ITD
MOLM-14	26.0 ± 7.7	17.9 ± 6.5	1.5 ± 0.6	wt	ITD
NB4	15.6 ± 4.3	22.5 ± 0.9	0.7 ± 0.2	mut	wt

Endogenous protein levels of Bcl-2 and Bax were determined by intracellular staining with labeled antibodies followed by flow cytometric analyses. Semi-quantitative measures of protein levels given as mean fluorescence intensity (MFI) were used to calculate the Bcl-2/Bax ratios. The results are based on three separate experiments performed in triplicates, with error bars representing standard error of mean. The genetic status of the prognostic markers *TP53 *and *FLT3 *have been reported by others [25-27].

A similar variation in khat and CPT sensitivity was observed when using nuclear morphology to assess cytotoxicity (Fig. 1B), although with some differences. Whereas the WST-1 viability/proliferation assay indicated that khat-treated MV-4-11 cells possessed 43.5 ± 10.8% (SEM) viability/proliferation as compared to controls, this cell line appeared relatively resistant to khat-induced changes in nuclear morphology (90.4 ± 7.0% (SEM) normal nuclei as compared to controls). NB4 was approximately as sensitive to khat as MOLM-13 and -14 based on the WST-1 assay (6.5 ± 3.9% (SEM) viability/proliferation as compared to controls), while it exhibited an intermediate level of sensitivity based on morphology (48.6 ± 7.2% (SEM) normal nuclei as compared to controls).

### Khat and CPT treatment induced distinct ultrastructural features

**Figure 2:**
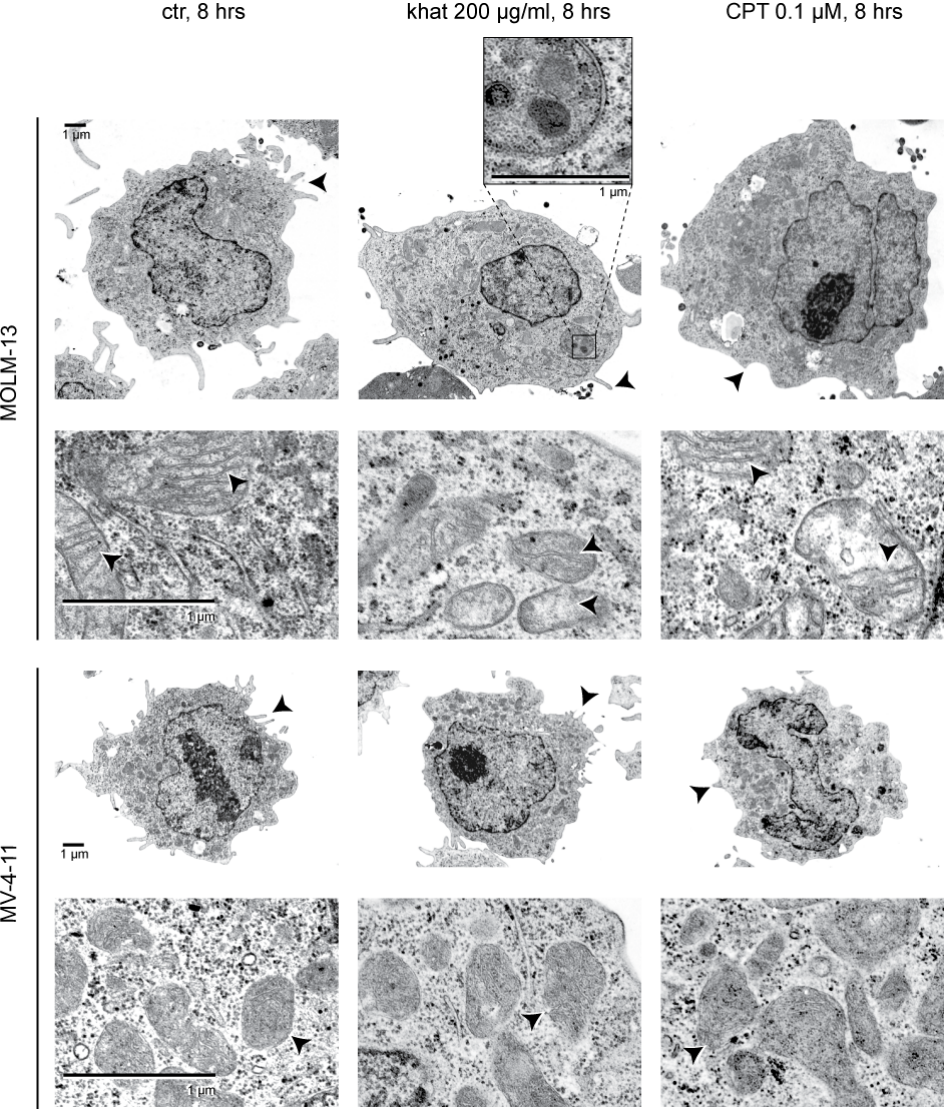
**Khat and CPT induced distinct ultrastructural features in MOLM-13 and MV-4-11**. MOLM-13 and MV-4-11 cells were exposed to 200 μg/ml khat and 0.1 μM CPT for 8 hours and prepared for transmission electron microscopy, see Methods for experimental details. Arrows indicate different ultrastructural features of microvilli and mitochondria. A double-membrane autophagosome is enlarged in the insert. The cells selected for this figure were either apparently unaffected or in early phases of cell death. Images of cells in late cell death are not shown.

To elucidate the mechanisms underlying khat- and CPT-induced cell death, the khat- sensitive MOLM-13 cell line and the partly khat-resistant MV-4-11 cell line were examined by transmission electron microscopy (TEM; Fig. 2). The lower CPT dose (0.1 μM) was chosen in this experiment since it caused the same level of toxicity in the two cell lines (Fig. 1B).

The mitochondria in untreated MV-4-11 cells appeared smaller, with less defined cristae compared to the mitochondria in untreated MOLM-13 cells. The most profound effect of khat observed was damage to the inner mitochondrial membrane in MOLM-13 cells. The affected mitochondria contained fewer cristae (1.9 ± 0.9 (SEM) cristae per mitochondria) compared to mitochondria in control cells (7.4 ± 1.3), suggesting that the cristae had been subjected to degradation. Khat treatment appeared to trigger autophagosome formation in MOLM-13 cells (Fig. 2, insert). The mitochondrial inner membranes in CPT-exposed MOLM-13 cells were partly affected (3.7 ± 1.0 (SEM) cristae pr mitochondria), while mitochondria in MV-4-11 seemed normal. Both khat and CPT caused involution of the endoplasmic reticulum, aggregation of ribosomes and reduced numbers of microvilli. MOLM-13 cells in late phases of khat-induced cell death were characterized by extensive cytosolic vacuolization and chromatin margination, while CPT mediated less prominent vacuolization and induced nuclear fragmentation (data not shown). MV-4-11 cells did not display ultrastructural changes following khat treatment, supporting the relative khat-resistance of this cell line. The observations based on TEM of khat- and CPT-treated MOLM-13 cells were scored and are summarized in Table 2.

**Table 2:** Summary of structural characteristics of MOLM-13

**Structural characteristics**:	control	khat	CPT
Normal mitochondria	+ + +	+	+ +(+)
Presence of ER	+ + +	+ +	+ +
Ribosomal clustering	-	+	+
Presence of microvilli	+ + +	+	+
Cytosolic vacuolization*	+	+ + +	+ +
Nuclear margination*	-	+++	-
Nuclear fragmentation*	-	+	+ + +

* scores based on khat/CPT treated cells in late phases of cell death

Based on TEM pictures different structural characteristics of MOLM-13 cells following khat (200 μg/ml) and CPT (0.1 μM) treatment were compared to controls and scored (n = 60).

### Khat induced impaired mitochondrial respiration in MOLM-13 and MV-4-11

**Figure 3:**
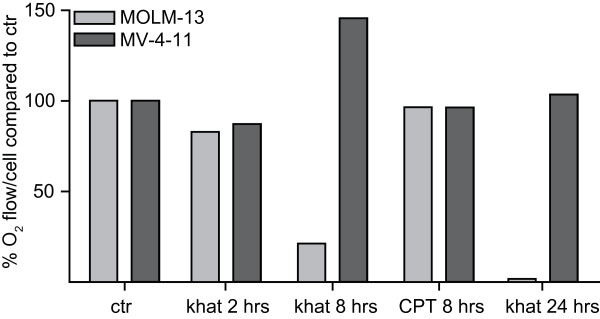
**Mitochondrial respiration was impaired in MOLM-13 while only temporary affected in MV-4-11**. MOLM-13 and MV-4-11 cells were exposed to 200 μg/ml khat and 0.1 μM CPT for the indicated hours and O_2 _consumption measured by high-resolution respirometry. Cellular O_2 _was calculated from the recorded data as the time derivative of the oxygen content in the chamber, and results presented as percentages O_2_consumption per cell in treated cells as compared to untreated controls. This figure shows the results from one typical experiment.

Based on the TEM observations and the partly diverging results from the viability/proliferation WST-1 assay and the nuclear morphology assay (Fig. 1), we decided to probe mitochondrial function in MOLM-13 and MV-4-11 in more detail. Mitochondrial routine respiration was assessed by measurements of cellular oxygen consumption rates after treatment with khat and 0.1 μM CPT. Mitochondrial respiration was slightly reduced in both MOLM-13 and MV-4-11 after 2 hrs of khat treatment, the effect being more pronounced in MOLM-13 cells (Fig. 3). After 8 hrs of khat exposure the mitochondrial routine respiration was significantly lowered in MOLM-13, consistent with the WST-1 assay (Fig. 1A). In contrast to MOLM-13, the respiration appeared restored in MV-4-11 after 8 hrs of khat treatment. After 24 hrs, khat-exposed MOLM-13 cells were no longer viable based on morphological examination and measurements of mitochondrial respiratory activity. The MV-4-11 cells, however, appeared morphologically normal and mitochondrial respiration in khat-treated cells was identical to respiration in the controls. CPT treatment for 8 hrs did not inhibit the mitochondrial routine respiration in MOLM-13 or MV-4-11 cells, even if inspection of nuclear morphology demonstrated induction of approximately 50% cell death (Fig. 1B). In general, the observed effects on routine respiratory rates paralleled those of maximal respiratory rates (data not shown).

The data indicated that khat treatment caused detrimental impairment of mitochondrial function in MOLM-13 cells, whereas the effect was minor and transient in MV-4-11 cells.

### Endogenous Bcl-2 levels and Bcl-2/Bax ratios did not correlate with cellular khat-sensitivity

Since khat affected mitochondrial structure and function, we hypothesized a role for the Bcl-2 family of proteins in khat-sensitivity. Endogenous Bcl-2 and Bax levels were assessed in the panel of AML cell lines using flow cytometry. Repeated measurements (n = 3) of Bcl-2 and Bax based on mean fluorescence intensity (MFI) were used to determine semi quantitative protein levels and to calculate Bcl-2/Bax ratios (Table 1).

However, no correlations were found between Bcl-2 and/or Bax levels and the observed khat- and CPT-sensitivities (Fig. 1A and 1B). The genetic status of the prognostic factors *TP53*, *FLT3 *and karyotype [25-27] did not determine cellular responsiveness to khat or CPT. This suggested other pathways and mechanisms as more important in determining cell death thresholds.

### Bcl-2 over-expression protected against CPT and partly against khat-induced death

**Figure 4:**
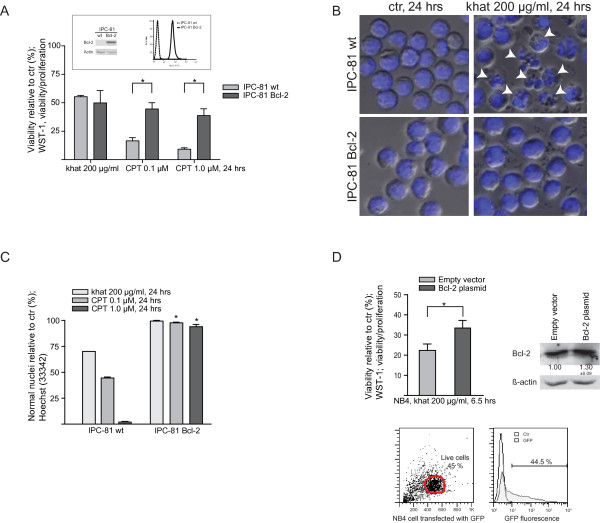
**Over-expression of Bcl-2 provided protection against CPT while only partly inhibiting khat-induced cell death**. IPC-81 parental and IPC-81 Bcl-2 rat leukemia cell lines were exposed 200 μg/ml khat and 0.1 μM CPT for 24 hrs and evaluated for toxic effects. **(A) **Effects on cell viability/proliferation were assessed using the WST-1 assay. The results were collected by measuring absorbance (450 nm - 620 nm) and are presented as: [(absorbance treated cells)/(absorbance control cells)] × 100. The figures in the insert demonstrate the different Bcl-2 protein levels in the two IPC-81 cell lines based on Western blotting and flow cytometry. The differences in CPT sensitivity between IPC-81 parental and IPC-81 Bcl-2 were statistical significant as indicated by the asterisk (p < 0.05). **(B) **The pictures of Hoechst stained IPC-81 parental and IPC-81 Bcl-2 cells following treatment with khat shows that khat only affected the IPC-81 parental cells. The arrows indicate the presence of cell fragmentation. **(C) **Effects on nuclear morphology were determined following Hoechst staining using epifluorescent microscopy. The results are presented as percentages cells with normal nuclear morphology as compared to controls. The differences in CPT sensitivity between IPC-81 parental and IPC-81 Bcl-2 were statistical significant (p < 0.05). **(D) **The NB4 cell line was transiently transfected with either an empty vector, a GFP-encoding control plasmid or a Bcl-2 plasmid and exposed to khat (200 μg/ml, 6.5 hrs) 24 hrs after transfection. Effects on cell viability/proliferation were assessed using the WST-1 assay showing a statistical significant protection against khat in the Bcl-2 transfected cells (p = 0.05).

To determine if khat- and CPT-sensitivity could be modulated by Bcl-2 levels within the same cell line, the rat acute promyelocytic cell line IPC-81 parental and its clone IPC-81 Bcl-2 were exposed to the two treatments. The IPC-81 Bcl-2 clone is stably transduced to express a Bcl-2 gene of human origin and has been reported to be resistant to pro-apoptotic stimuli [28]. The cell lines were exposed to 200 μg/ml khat and 0.1 μM CPT and toxic effects were evaluated after 24 hrs using the viability/proliferation WST-1 assay and examination of nuclear morphology (Fig. 4A and 4B). The viability/proliferation assay demonstrated that khat-exposed IPC-81 parental and IPC-81 Bcl-2 possessed approximately 50% lower viability compared to untreated controls. In contrast, the toxic effects of CPT were significantly inhibited in the *BCL-2 *transduced clone compared to the parental cell line (Fig. 4A).

Inspection of khat-treated samples revealed that the IPC-81 parental included apoptotic cells and relatively large amounts of cell debris, which contrasted with the IPC-81 Bcl-2 clone (Fig. 4B). It was estimated that at least 30% of the IPC-81 parental cells had died following khat treatment, whereas the Bcl-2 over-expressing clone appeared approximately resistant. CPT-induced cell death was significantly inhibited by Bcl-2 over-expression (Fig. 4C).

To validate the results obtained with the IPC-81 cells, the human promyelocytic NB4 cell line was transiently transfected with either an empty control plasmid, a GFP-encoding control plasmid (45% post-transfection viability at 24 hrs, transfection efficiency > 40%; Fig. 4D) or a Bcl-2 encoding plasmid prior to khat exposure. Western blotting demonstrated an increase in Bcl-2 protein level by 30% in the Bcl-2 plasmid transfected cells compared to the control cells (Fig. 4D). The WST-1 viability/proliferation assay indicated that enforced Bcl-2 expression in NB4 cells provided a modest, but significant, protection against khat-induced cell death (Fig. 4D). These results lead us to further examine the role of Bcl-2 family proteins.

### Khat induced Mcl-1 reduction and caspase-8 activation in MOLM-13 cells

**Figure 5:**
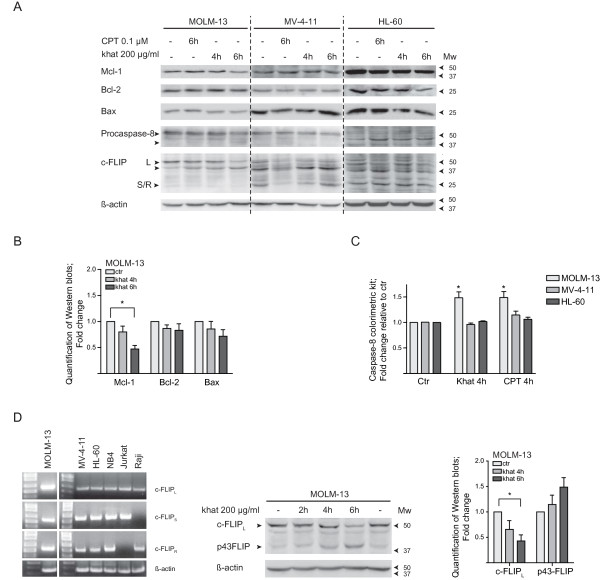
**Khat induced c-FLIP cleavage and Mcl-1 attenuation in khat sensitive MOLM-13**. **(A) **MOLM-13, MV-4-11 and HL-60 were exposed to 200 μg/ml khat and 0.1 μM CPT for 4 and/or 6 hrs and evaluated for selected protein changes by Western blotting techniques. **(B) **Selected results for MOLM-13 were quantified using the KODAK Image Station 2000R software; the values normalized to the actin loading control and fold changes calculated. The asterisk indicates a significant reduction in Mcl-1 levels (p < 0.05). **(C) **MOLM-13, MV-4-11 and HL-60 were exposed to 200 μg/ml khat and 0.1 μM CPT for 4 hrs and caspase-8 activity evaluated using a Caspase-8 colorimetric kit. Fold change in enzymatic activity was determined by comparing the khat- and CPT-exposed samples to untreated controls. **(D) **Reverse transcription PCR was used to determine the expression of different c-FLIP RNA isoforms in the leukemic cell lines tested. Jurkat and Raji cells were utilized as controls since they are known to express the cFLIP_S _and c-FLIP_R _isoforms, respectively. β-Actin served as loading control. MOLM-13 was exposed to 200 μg/ml khat for 2, 4 and 6 hrs and evaluated for c-FLIP_L _cleavage by Western blotting techniques. The results were quantified, normalized to the actin loading control and fold changes calculated. The asterisk indicates a significant reduction in c-FLIP_L _levels (p < 0.05).

Protein levels of Bcl-2 family members were analyzed by standard Western blotting techniques following treatment with khat and 0.1 μM CPT. The experiments and analyses included the relatively khat-resistant MV-4-11 cell line, in addition to the khat-sensitive MOLM-13, HL-60 (Fig. 5) and NB4 cell lines (data not shown).

A significant reduction was observed in anti-apoptotic Mcl-1 levels in MOLM-13 after 6 hrs of khat treatment (p < 0.05), whereas Bcl-2 and Bax levels were not significantly altered (Fig. 5A and 5B). Both khat and CPT appeared to mediate cleavage-activation of procaspase-8 in the MOLM-13 cell line (Fig. 5A). Caspase-8 activity in MOLM-13 was further supported by results obtained with a Caspase-8 colorimetric kit, where a 1.47 ± 0.11 (SEM) fold-increase in caspase-8 activity was observed following khat exposure and a 1.48 ± 0.12 (SEM) fold-increase following CTP treatment, as compared to controls. In contrast, neither khat nor CPT was observed to mediate procaspase-8 activation in MV-4-11 and HL-60 (Fig. 5C). Attempts to inhibit khat-induced cell death in MOLM-13 and HL-60 were made by treating the cells with a specific inhibitor of caspase-8; Z-IETD-FMK, prior to khat addition. However, a significant protective effect could not be detected from this pre-treatment based on the WST-1 assay and nuclear morphology (results not shown).

### Khat and CPT induced c-FLIP_L _cleavage

A statistical significant decrease (0.41 ± 0.09 (SEM) fold change as compared to controls after 6 hrs) in c-FLIP_L _levels was observed in khat-treated MOLM-13 cells (Fig. 5A and 5D) whereas no significant changes were detected in MV-4-11 and HL-60. In accordance with previous reports, c-FLIP appeared to be cleaved by CPT treatment in MOLM-13, MV-4-11 and HL-60 [20] (Fig. 5A). Attempts to sensitize the cell lines to khat-induced cell death by transfection with siRNA against c-FLIP were made, but increased susceptibility was not demonstrated (results not shown).

The Western blots of c-FLIP expression in MOLM-13, MV-4-11 and HL-60 displayed different patterns of protein bands, and may reflect differential expression of different c-FLIP isoforms in the cell lines (Fig. 5A) [17,18]. However, RT-PCR of the c-FLIP isoforms (c-FLIP_L_, c-FLIP_S _and c-FLIP_R_) indicated that the various cell lines used expressed all three isoforms [29] (Fig. 5D).

## Discussion

The MOLM-13 cell line and the MV-4-11 cell line represent a monocytic acute myeloid leukemia and acute biphenotypic leukemia, respectively. The cell lines share the characteristics of wild type *TP53 *and Flt3 length mutations/internal tandem duplications (ITDs), representing molecular characteristics with prognostic value in AML patients.

When comparing selected AML cell lines, the MOLM-13 cell line was observed to be one of the most khat-sensitive, whereas MV-4-11 represented the most khat-resistant. The difference in sensitivity was detected both with nuclear morphology analyses and with the WST-1 assay (Fig. 1). A possible explanation for the more accentuated effects displayed in the MV4-11 cells by the WST-1 assay (Fig. 1A) lies in the reliance of the WST-1 assay on enzymatic complex II activity of the mitochondrial respiratory chain [30]. The reduced viability/proliferation indicated by the WST-1 assay was in agreement with the impaired mitochondrial routine respiration initially detected in both MOLM-13 and MV-4-11 (Fig. 3). In contrast to MOLM-13, MV-4-11 recovered from the khat-induced mitochondrial impairment after 8 hrs of khat exposure. CPT exposure did not significantly affect mitochondrial routine respiration in neither MOLM-13 nor MV-4-11. This suggested that khat and CPT caused cell death through different mechanisms.

Complex I and III of the respiratory chain are the main ROS production sites in the cell [31] and mitochondrial dysfunction may lead to excessive ROS levels inducing damage to DNA, proteins and membranes [16]. We previously reported that khat induced ROS in normal oral keratinocytes and fibroblasts *in vitro *[2] and recently that khat affected mitochondrial inner transmembrane potential in these primary cells [32]. Another study suggests that khat and the alkaloid fraction produce oxidative stress in rats by reducing the levels of free radical scavenging enzymes and glucose, leading to increased ROS and toxicity [33]. These findings, together with the khat-mediated mitochondrial damage reported in this study, suggest that ROS could be involved in khat-mediated toxicity in AML cell lines. Interestingly, it has been reported that treatment of leukemic cells with inhibitors of mitochondrial function lead to increased ROS and sensitization of the cells to drug-induced apoptosis [34].

Khat-treated MOLM-13 cells in late phases of cell death displayed extensive vacuolization of the cytoplasm indicating increased autophagy [12]. Untreated MOLM-13 cells contained abundant lysosomes while autophagosomes were observed in khat-exposed cells (Fig. 2), further supporting involvement of the autophagosomal-lysosomal compartment in khat-induced cell death [35]. It is possible that autophagy is triggered in MOLM-13 as a consequence of khat-induced mitochondrial damage and concomitant ATP deprivation.

When investigating whether endogenous Bcl-2 levels affected khat- and CPT- sensitivities using the IPC-81 cell lines, Bcl-2 over-expression was observed to protect against khat-mediated death. As previously reported, CPT-induced cell death was inhibited by Bcl-2 over-expression [36,37] (Fig. 4). However, endogenous Bcl-2/Bax ratios could not be correlated to the different khat- and CPT-sensitivities in the AML cell line panel (Table 1). This suggested other molecular characteristics as more important determinants for cellular responsiveness to khat and CPT.

Khat treatment induced significant down-regulation of the anti-apoptotic Mcl-1 protein in MOLM-13 (Fig. 5A and 5B). Neutralization of Mcl-1 has been indicated to play a central role when targeting Bcl-2 family members in apoptosis-induction. Mcl-1 also connects the receptor- and mitochondria-mediated cell death pathways by antagonizing the caspase-8 cleavage product tBid, which may induce mitochondrial cytochrome C release [38,39]. It is possible that khat-mediated procaspase-8 activation and Mcl-1 down-regulation act in conjunction in cell death-induction in MOLM-13. Mcl-1 has also been shown to interact with Beclin-1, a Bcl-2 interacting protein that promotes autophagy [40]. BH3-only pro-apoptotic proteins have been shown to mediate autophagy by competitively disrupting the binding of Beclin-1 to anti-apoptotic Bcl-2 proteins [41].

When analyzing components of receptor-mediated cell death, a significant reduction in c-FLIP_L _levels was detected in khat-treated MOLM-13 cells. The reduction appeared to result from cleavage of this full length isoform to the p43-FLIP fragment (Fig. 5A and 5D). Procaspase-8 and active caspase-8 have been shown to exhibit different specificities when catalyzing c-FLIP cleavage [42]. Khat-induced generation of p43-FLIP indicated the presence of active caspase-8, suggesting involvement of death receptor signaling [1]. In agreement with this, procaspase-8 activation was observed to be mediated by both khat and CPT in the MOLM-13 cell line (Fig. 5A and 5C).

CPT was shown to induce c-FLIP_L _cleavage in MOLM-13 and cleavage reactions were also indicated in MV-4-11 and HL-60 (Fig. 5A). Reduced c-FLIP expression and increased susceptibility to death receptor-induced apoptosis has been demonstrated for CPT and drugs like cisplatin, doxorubicin and trichostatin A [20,43]. Transfection experiments were performed with siRNA against c-FLIP, but sensitization to khat-induced cell death was not observed in MOLM-13, MV-4-11 or HL-60 (results not shown). c-FLIP down-regulation has been reported to occur in conjunction with ROS generation [22,44,45], and ROS has as mentioned been implicated in *in vitro *and *in vivo *studies on khat toxicity [2,33].

## Conclusion

We have shown that a botanical khat extract induced cell death in a subset of AML cell lines, indicating distinct molecular mechanisms involved as compared to CPT-induced apoptosis. Khat was shown to target mitochondrial structure and function, which may trigger enhanced autophagy and induce further cellular damage. Future studies should identify the pro-cell death components in khat and evaluate the potential of these substances within experimental cancer therapy.

## Methods

### Khat (Catha edulis Forsk.) extract preparation and analysis

Khat (asili cultivate) samples from the Meru district in Kenya were transported at 4°C under moist conditions. The material was extracted within 48 hrs of harvest using methanol extraction as previously described [1]. The methanol extract was dried using a rotary evaporator and the semi solid residue dissolved at a concentration of 0.2 g/ml dimethylsulphoxide (DMSO) (Sigma, St. Louis, MO, USA) for storage in -80°C. The DMSO stock solution was diluted 1:10 in cell culture medium with 10% fetal bovine serum (FBS) and antibiotics. The dilution step resulted in precipitate formation [1]. In this study the precipitate was removed by centrifugation (10 000 × g_av_., 15 min, 4°C), and the supernatant was further diluted 1:100 in cell culture medium giving a final dilution of 1:1000, corresponding to 200 μg/ml based on the DMSO stock concentration. The DMSO concentration in experimental cell cultures was 0.1% and control cells were added an equivalent amount of DMSO. Cells were exposed to the khat extract for different periods of time (4, 6, 8 and 24 hrs) before being analyzed for changes in viability/proliferation, nuclear morphology, mitochondrial respiration and protein levels.

The concentrations of cathinone, cathine and norephedrine in the khat extract were determined using high pressure liquid chromatography and mass spectrometry (HPLC-MS-MS). The khat extract was diluted 1:100 in water containing 0.1% formic acid and separated using an Ace 3 Phenyl column (2.1 × 50 mm, 3 μm) (Advanced Chromatography Technologies). The alkaloids were eluted with a gradient of methanol and 0.1% formic acid with 5 mM ammonium formiate in H_2_O. The MS analysis was carried out using positive electrospray ionization on an Agilent G6410A QQQ Mass Spectrometer (Agilent Technologies Inc., CA, USA). The measured alkaloid concentrations were as follows: S-(-)-cathinone: 2.5, (1S, 2S)-(-)-norpseudoephedrine (cathine) 3.0 and (1R, 2S)-(-)-norephedrine: 0.3; all values presented as mg/ml.

### Cultivation and characteristics of AML cell lines

The biphenotypic cell line MV-4-11 was purchased from ATCC (American Type Culture Collection, Manassas, VA, USA) and cultured in IMDM (BioWhittaker, Cambrex Bio Science, Verviers, Belgium) with 10% FBS, 2 mM L-glutamine, 100 IU/ml penicillin and 100 μg/ml streptomycin (Gibco, Grand Island, NY, USA). The promyelocytic HL-60 was purchased from DSMZ (Deutsche Sammlung von Mikroorganismen und Zellkulturen, Braunschweig, Germany); the monocytic MOLM-13 and MOLM-14 were a generous gift from Dr. Kunzo Orita, Okayama, Japan; the promyelocytic NB4 was a generous gift from Dr. Michel Lanotte, Hôpital St. Louis, Paris, France; all cell lines were cultured in RPMI 1640 (Sigma, St. Louis, MO, USA) with 10% FBS, L-glutamine and antibiotics. The IPC-81 cell lines, also a gift from Dr. Michel Lanotte, Paris, were cultured in RPMI 1640 (Sigma, St. Louis, MO, USA) with 10% horse serum, L-glutamine and antibiotics. Jurkat and Raji cell lines were cultivated in RPMI 1640 with 10% FBS, L-glutamine and antibiotics. The Jurkat cells were purchased from ATCC while the Raji cells were a kind gift from Torbjørn Hansen, The Gade Institute, University of Bergen, Norway. Cells were routinely screened for mycoplasma and maintained in a humidified atmosphere at 37°C with 5% CO_2_. The cell lines were seeded at a concentration of 200 000 cells/ml when used in experiments. Molecular characteristics of the cell lines are shown in Table 1.

### Cell viability/proliferation assay

Viability/proliferation was determined using the Cell Proliferation Reagent WST-1 (Roche Applied Science) according to the manufacturer's protocol. Aliquots (90 μl) of cells (200 000 cells/ml) were added 10 μl WST-1 four hours after initiation of the experiments. The cells were treated for 4 more hours and the results collected using a Tecan Infinite 200 microplate reader and Magellan software (version 6) (Tecan Trading AG, Switzerland). The results are presented as percentage viability/proliferation of treated cells relative to controls, using the formula: [(abs. treated cells)/(abs. control cells)] × 100.

### Cell death determination based on nuclear morphology

Cell death was determined as described by Gjertsen et al. [46] by microscopy of cell aliquots (50 μl) fixed in nutrient media containing 4% formaldehyde with 10 μg/ml of the DNA-specific fluorochrome, bisbenzimide (Hoechst 33342; Calbiochem, San Diego, CA, USA). Nuclear morphology was examined under epifluorescent microscopy using a Leica IRB inverse microscope.

### Intracellular staining and flow cytometry

Intracellular staining of Bcl-2 and Bax with conjugated antibodies was performed according to procedures previously described [47]. Samples of 3 × 10^6 ^cells were washed with ice cold NaCl (9 mg/ml), fixed with 2% paraformaldehyde (Sigma-Aldrich, St. Louis, MO, USA) and permeabilized with cold methanol. Cells were washed with 1% bovine serum albumine (BSA) in phosphate buffered saline (PBS) and antibodies added. Cells were labeled with FITC-conjugated hamster anti-human Bcl-2 (6C8), isotype control (Ha4/8) (both from FITC-conjugated Bcl-2 Antibody Reagent Set, BD Pharmingen, San Diego, CA, USA) or PE-labeled anti-human Bax (clone 2D2, Santa Cruz Biotechnology, CA, USA). The samples were washed and resuspended in 1% BSA in PBS and subjected to flow cytometry.

Flow cytometric data were collected on a FACS Calibur (Becton Dickinson Immunocytometry Systems, San Jose, CA, USA) and analyzed using FlowJo software (Tree Star, Inc., Ashland, OR, USA). A semi-quantitative measure of protein levels, given as mean fluorescence intensity (MFI), was calculated by subtracting the unspecific binding of the isotype control from the bound conjugated antibody.

### Transmission electron microscopy

Cells were fixed in 0.1 M Na-cacodylate buffer, pH 7.4 containing 2% glutaraldehyde. Samples were rinsed with buffer and post-fixed in 1% osmium tetroxide. The specimens were dehydrated using graded ethanol and embedded in epoxy resin, and ultra-thin sections double stained with uranyl acetate and lead citrate [48]. Specimens were examined with electron microscope (JEOL 1230, Jeol Ltd., Tokyo, Japan) and the micrographs processed using an AGFA Arcus II scanner and Adobe Photoshop 7.0.1 software.

### Oxygen consumption rates

O_2 _consumption was determined by high-resolution respirometry using an Oroboros Oxygraph-2k instrument (OROBOROS^® ^INSTRUMENTS GmbH, Innsbruck, Austria). Cells were seeded at a total of 4 × 10^6 ^cells and harvested after different khat and CPT treatments. The cells were spun down at 1000 rpm for 4 minutes and resuspended in MIR05 buffer (Oroboros lab). The respiration experiments were conducted at 37°C in MIR05 buffer. A standard protocol using malate (2 mM), glutamate (10 mM), oligomycin (2 μg/ml), FCCP (0.45 μM), succinate (10 mM), digitonin (3.68 μM), rotenone (0.5 μM) and antimycin A (2.5 μM) was used for each measurement. Cellular O_2 _was calculated from the recorded data as the time derivative of the oxygen content in the chamber, O_2 _concentrations were calculated using DatLab software (Oroboros Instruments).

### Transfection and siRNA

Plasmids (5 μg) were introduced into 2 × 10^6 ^NB4 cells using the Cell Line Nucleofector™ Kit V from Amaxa (Amaxa GmbH, Cologne, Germany) and the Amaxa Nucleofector™ Device. The bill neo control plasmid and the bill neo Bcl-2 expression plasmid were generous gifts from Dr. Timothy J. McDonnell, M.D. Anderson Cancer Center, Houston, USA [49]. The transfected cells were challenged with khat and CPT 24 hrs after transfection. Toxic effects mediated by khat/CPT were evaluated using the WST-1 assay and nuclear morphology.

Different concentrations (10 nM, 50 nM and 100 nM) of *Silencer*^® ^Select siRNA against c-FLIP and control siRNA (Ambion Inc., Applied Biosystems Inc., Foster City, CA, USA) were transfected into MOLM-13, HL-60 and MV-4-11 cells using the optimized protocols found at http://www.lonzabio.com. The transfected cells were exposed to khat 24 hrs after transfection and toxicity evaluated using the WST-1 assay and nuclear morphology.

### Western blotting

Cells (4 × 10^6 ^pr experimental condition) were washed with ice cold NaCl (9 mg/ml) and subjected to gel electrophoresis (12.5% gels) and Western blotting as previously described [50]. The membranes were probed with antibodies against Bad (610391) from BD Biosciences (Franklin Lakes, NJ, USA) and procaspase-8 (sc-7890), c-FLIP_S/L _(sc-5276), Bcl-2 (sc-783), Bax (sc-20067), Mcl-1 (sc-12756), Noxa (sc-30209); all purchased from Santa Cruz Biotechnology, CA, USA. Anti-beta-actin (mAbcam no. 8226; Abcam Inc., Cambridge, MA, USA) was used as loading control. IgG secondary antibodies conjugated to Horseradish Peroxidase (Jackson ImmunoResearch, West Grove, PA, USA) were detected by enhanced chemiluminescence (Supersignal^® ^West Pico or Supersignal^® ^West Femto; Pierce Biotechnology, Rockford, IL, USA) and visualized/analyzed using a KODAK Image Station 2000R (Eastman Kodak Company, Rochester, NY, USA).

### Caspase-8 colorimetric kit and inhibition

Caspase-8 activation was assessed using the Caspase-8 colorimetric kit from R&D Systems Inc. (Minneapolis, MN, USA). The protocol provided by the manufacturer was followed with some adjustments.

Inhibition of caspase-8 activity was attempted by pre-treating the cells (200 000 cells/ml) with 10^-5 ^M and 10^-6 ^M Z-IETD-FMK (Sigma-Aldrich, St. Louis, MO, USA) for 15 minutes prior to khat addition (200 μg/ml). Toxic effects were evaluated with the WST-1 assay and nuclear morphology.

### RNA isolation and RT-PCR

Total RNA was isolated from 5 to 10 × 10^6 ^cells using the RNeasy kit (QIAGEN, Hilden, Germany) and cDNA was synthesized from 1 μg of total mRNA with the Cloned AMV First-Strand cDNA Synthesis Kit (Invitrogen Ltd., Paisley, UK). c-FLIP_L_, c-FLIP_S_, and c-FLIP_R _was amplified from cDNA using the HotStarTaq PCR kit from Qiagen with primers as described by Ueffing [29].

### Statistical analysis

Fold change data were based on quantification of Western blots using the KODAK Image Station 2000R software, and evaluated for statistical significance (p < 0.05) using Student's t test.

## Competing interests

The authors declare that they have no competing interests.

## Authors' contributions

TB and EAOD designed, performed experiments and wrote the manuscript. HRH and KJT contributed to the experiments in Figure 3. LW and JS performed experiments and analyzed data. KOF analyzed the khat extract. ACJ, OKV and BTG designed the study and wrote the manuscript. All authors have read and approved the final manuscript.
